# Dynamic fronto‐amygdalar interactions underlying emotion‐regulation deficits in women at higher weight

**DOI:** 10.1002/oby.23830

**Published:** 2023-08-06

**Authors:** Pablo Maturana‐Quijada, Trevor Steward, Nuria Vilarrasa, Romina Miranda‐Olivos, Susana Jiménez‐Murcia, Holly J. Carey, José‐Antonio Fernández‐Formoso, Fernando Guerrero‐Perez, Isabel Sánchez, Nuria Custal, Nuria Virgili, Rafael Lopez‐Urdiales, Carles Soriano‐Mas, Fernando Fernandez‐Aranda

**Affiliations:** ^1^ Psychiatry and Mental Health Group, Neuroscience Program Institut d’ Investigació Biomèdica de Bellvitge (IDIBELL) Barcelona Spain; ^2^ Melbourne School of Psychological Sciences, Faculty of Medicine, Dentistry and Health Sciences University of Melbourne Parkville Victoria Australia; ^3^ Melbourne Neuropsychiatry Centre, Department of Psychiatry, Faculty of Medicine, Dentistry and Health Sciences University of Melbourne Parkville Victoria Australia; ^4^ Department of Endocrinology and Nutrition Bellvitge University Hospital–IDIBELL Barcelona Spain; ^5^ CIBER Diabetes and Associated Metabolic Diseases (CIBERDEM) Instituto de Salud Carlos III Barcelona Spain; ^6^ Ciber Fisiopatología Obesidad y Nutrición (CIBERObn) Instituto Salud Carlos III Barcelona Spain; ^7^ Psychoneurobiology of Eating and Addictive Behaviors Group, Neuroscience Program Institut d' Investigacio Biomèdica de Bellvitge (IDIBELL) Barcelona Spain; ^8^ Department of Clinical Sciences, School of Medicine and Health Sciences University of Barcelona Barcelona Spain; ^9^ Clinical Psychology Unit Bellvitge University Hospital‐IDIBELL Barcelona Spain; ^10^ Ciber Salud Mental (CIBERSAM) Instituto Salud Carlos III Barcelona Spain; ^11^ Department of Social Psychology and Quantitative Psychology, School of Psychology University of Barcelona Barcelona Spain

## Abstract

**Objective:**

The regulation of negative emotions entails the modulation of subcortical regions, such as the amygdala, by prefrontal regions. There is preliminary evidence suggesting that individuals at higher weight may present with hypoactivity in prefrontal regulatory systems during emotional regulation, although the directionality of these pathways has not been tested. In this study, we compared fronto‐amygdalar effective connectivity during cognitive reappraisal as a function of BMI in 48 adult women with obesity and 54 control participants.

**Methods:**

Dynamic causal modeling and parametric empirical Bayes were used to map effective connectivity between the dorsomedial prefrontal cortex, orbitofrontal cortex, dorsolateral prefrontal cortex, and the amygdala.

**Results:**

Difficulty in Emotion Regulation Scale scores were higher in the obesity group compared with control participants (*p* < 0.001). A top‐down cortical model best explained our functional magnetic resonance imaging data (posterior probability = 86%). Participants at higher BMI were less effective at inhibiting activity in the amygdala via the orbitofrontal cortex and dorsomedial prefrontal cortex during reappraisal compared with those at lower BMI. In contrast, increased excitatory modulation of dorsolateral prefrontal cortex‐to‐amygdalar connectivity was found in participants at lower BMI.

**Conclusions:**

These findings support a framework involving alterations in fronto‐amygdalar connectivity contributing to difficulties in regulating negative affect in individuals at higher weight.

**Study Importance**

**What is already known?**

- Deficits in emotional regulation contribute to the onset and maintenance of higher weight.
- Individuals at higher weight are more likely to use food intake as a maladaptive emotion‐regulation strategy.
- Individuals at higher weight may present with hypoactivity in prefrontal regulatory systems during emotional regulation, though the directionality of these pathways has not been tested.

**What does this study add?**

- This study used dynamic causal modeling to determine how BMI impacts fronto‐amygdalar effective connectivity during cognitive reappraisal.
- We found strong evidence to support that higher BMI is associated with less effective inhibitory modulation of the amygdala by prefrontal control regions during reappraisal.

**How might these results change the direction of research or the focus of clinical practice?**

- This study represents a meaningful step forward in improving our understanding of how emotion‐regulation mechanisms are affected in women with higher BMI.
- Our findings uphold prevalent models of emotional regulation and support that altered neurobiological function contributes to the difficulties in adequately assessing and managing negative affective states at higher weight.
- Targeting emotion‐regulation processes may be beneficial for some at higher weight.

## INTRODUCTION

High body weight is thought to be maintained by hypothalamic signals influencing calorie intake, glucose metabolism, and energy expenditure, as well as by psychological factors [1]. Specifically, deficits in emotional regulation are thought to contribute to the onset and maintenance of higher weight as there is mounting evidence indicating that individuals at higher weight are more likely to use food intake as a maladaptive emotion‐regulation strategy [2].

Emotional regulation encompasses efforts through which people alter the experience and/or expression of their emotions [3]. Of these emotion‐regulation strategies, neuroimaging research has largely focused on cognitive reappraisal, which involves reinterpreting the meaning of affectively charged stimuli or events in terms that alter their emotional impact. Meta‐analyses on the neurobiological correlates of cognitive reappraisal in healthy control individuals have converged in demonstrating that the prefrontal cortex (PFC) modulates activity in regions associated with negative affect, such as the amygdala and the insula, with the amygdala being the subcortical region most found to be modulated during reappraisal [4, 5].

Two previous studies have examined the cognitive reappraisal of negative emotions in individuals at higher weight and with obesity using functional magnetic resonance imaging (fMRI). One study found that young adults with higher weight displayed increased functional connectivity between the right anterior insula and the dorsolateral and dorsomedial prefrontal cortices (dlPFC and dmPFC, respectively) during reappraisal [6]. Relatedly, another study identified that adult women with obesity presented with a decreased response in the ventromedial PFC when reappraising negative emotions [7], with ventromedial PFC activity levels during cognitive reappraisal being negatively correlated with self‐reported difficulties in emotional regulation. These studies, however, have examined task‐induced activations and functional connectivity during reappraisal, but no study thus far, to our knowledge, has modeled the causal dynamics of between‐region connectivity. Dynamic causal modeling (DCM) allows for testing causal excitatory and inhibitory connectivity by inferring the causal architecture of a network of regions (i.e., nodes) and estimating their “effective connectivity,” that is, the extent to which the activity of one region directly influences activity in other regions or the modulatory effects of a task on causal architecture [8]. DCM, therefore, can deliver more refined neurobiological models of emotion‐regulation alterations in individuals at higher weight and provide more informed insights on which regions and pathways are, for example, potentially well suited for focal stimulation.

Considering the robust evidence of emotional regulation‐modulating amygdala activity via the PFC, the present study aimed to model the causal architecture of fronto‐amygdalar interactions in women at higher weight and healthy control individuals during cognitive reappraisal using DCM. Specifically, we sought to test how body mass index (BMI) accounted for individual differences in the modulatory effects of cognitive reappraisal on prefrontal‐amygdala pathway dynamics. We hypothesized that we would observe decreased prefrontal modulation of amygdala activity as a function of increased BMI.

## METHODS

### Participants

Our sample was made up of 102 adult women with BMI ranging between 18.5 and 60. A total of 48 of these participants had obesity (BMI > 30) and were recruited from the Bariatric and Metabolic Surgery Unit and the Endocrinology and Nutrition Unit at Bellvitge University Hospital in Barcelona, Spain. The recruitment period was between 2016 and 2021. A total of 44 control participants without obesity (BMI < 30) were recruited from the local community. All participants underwent the Mini‐International Neuropsychiatric Interview [9] with staff psychologists from the Department of Psychiatry at Bellvitge University Hospital. Inclusion criteria included being female and being between 18 and 55 years of age. Exclusion criteria included the presence of an intellectual disability, the presence or history of neurological or major medical disorders, the presence or history of an eating disorder or other psychiatric conditions (i.e., psychotic disorders, bipolar disorder, substance dependence, or mood disorders), binge‐eating episodes, or the presence of MRI contraindications. Participants without obesity were asked to report maximum lifetime BMI, and those who endorsed having had obesity (BMI > 30) were excluded from the study. Participants received compensation for participating in the study.

The present study was carried out in accordance with the latest version of the Declaration of Helsinki. The Bellvitge University Hospital Clinical Research Ethics Committee and Institutional Review Board approved the study (PR146/14). Signed informed consent was obtained from all participants.

### Clinical measures

All participants completed the Difficulties in Emotion Regulation Scale (DERS) [10]. This 36‐item self‐report measure assesses emotion‐regulation difficulties using six separate subscales. Higher scores indicate greater emotion‐regulation impairment.

### Anthropometric measures

A Tanita BC‐420MA was used to assess body composition and calculate BMI. This noninvasive validated device uses bioelectrical impedance analysis to measure weight and body composition variables (i.e., body fat percentage) [11]. Height was measured via stadiometer.

### fMRI cognitive reappraisal paradigm

**FIGURE 1:**
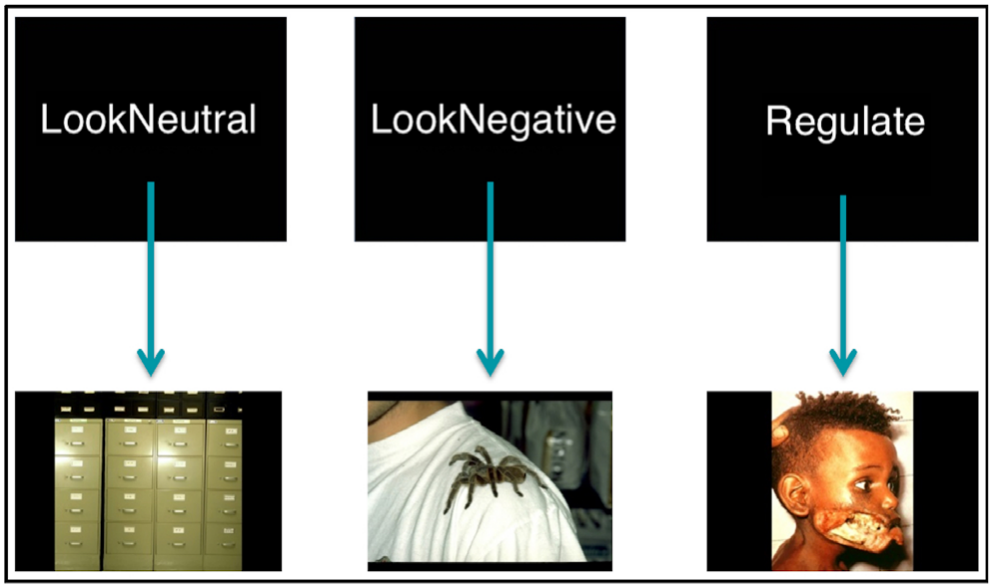
Functional MRI task example images for LookNeutral, LookNegative, and Regulate conditions. Participants were presented with neutral and negative images and were instructed to either (1) LookNeutral or passively observe neutral images; (2) LookNegative or maintain emotions elicited by negative images; or (3) Regulate or reappraise negative emotions elicited by negative images. [Color figure can be viewed at wileyonlinelibrary.com]

A modified version of the cognitive reappraisal task [12] was used. It included three conditions “LookNeutral,” “LookNegative,” and “Regulate,” which were presented following a block design. Blocks were presented displaying neutral or negative picture stimuli (Figure 1) that participants were instructed to (1) LookNeutral (passively observe neutral images); (2) LookNegative (actively sustain the emotions elicited by the negative images); or (3) Regulate (reappraise and reduce the intensity of negative emotions by means of previously trained cognitive reappraisal techniques). Further descriptions of the task have been reported elsewhere [7], and they can be found in the online Supporting Information.

### Statistical analysis of behavioral data

Statistical analysis for clinical and behavioral data was carried out with SPSS Statistics version 21 (IBM Corp.). Interactions between in‐scanner ratings for each condition (LookNeutral, LookNegative, and Regulate) and group were evaluated using repeated‐measures ANOVA to behaviorally confirm participants' ability to engage in the task. We used independent sample *t* tests for between‐group comparisons as well as Pearson correlations for evaluating linear associations between variables. Shapiro–Wilk tests were performed to confirm normality of the variables of interest, and we checked for the presence of outliers.

### fMRI acquisition and preprocessing

Neuroimaging data were acquired using a 3.0‐T Philips Ingenia MRI scanner equipped with a 32‐channel head coil. During acquisition, T2‐weighted echo‐planar imaging was obtained (repetition time [TR] = 2000 milliseconds, echo time [TE] = 25 milliseconds, field of view = 228 × 228 mm, 76 × 76 matrix, flip angle = 90°, 40 axial sections of 3‐mm thickness, 234 scans). A sagittal three‐dimensional T1‐weighted turbo‐gradient‐echo sequence (233 sections, TR = 10.63 milliseconds, TE = 4.91 milliseconds, flip angle = 8°, field of view = 240 × 225, 1‐mm^3^ voxels) was also obtained for anatomical reference.

Prior to preprocessing, we applied an artifact reduction method using the BrainWavelet toolbox [13]. Next, using MATLAB version 9.3 (R2017b, The MathWorks Inc.) and the MATLAB‐based CONN‐fMRI Functional Connectivity toolbox version 18.4 [14], implemented in SPM12, functional images were aligned to the first volume of the time series using a six‐parameter rigid body spatial transformation and a least‐squares minimization in combination with an unwarping algorithm aimed at correcting motion and motion‐related distortions. Slice‐timing correction was then applied. Artifact detection/identification toolbox (ART)‐based automatic volume outlier detection was also run for later volume scrubbing. Functional and structural images were subjected to simultaneous gray matter, white matter, and cerebrospinal fluid segmentation, and a bias correction was performed to remove smoothly varying intensity differences across images. These image segments were subsequently spatially normalized through nonlinear transformations to the Montreal Neurological Institute stereotactic space, and images were resliced to a 2‐mm isotropic resolution. Finally, images were smoothed with an isotropic gaussian kernel of 8‐mm full width at half maximum.

After preprocessing, data were denoised using temporal despiking, regressing out confounding factors (i.e., effect of blood oxygen level‐dependent [BOLD] signal small ramping effects at the beginning of each scan session and the six rigid realignment parameters, as well as their first‐order derivatives), controlling for total gray matter signal, linear detrending, the ART scrubbing protocol, and band‐pass filtering (0.008–0.09 Hz). The ART scrubbing protocol regressed out the effect of outlier volumes whose signal intensity deviated >5 SDs from the mean time series signal intensity or that showed evidence of displacement superior to 0.9 mm in relation to the preceding volume. Two participants were excluded because of excessive movement. Further information on fMRI acquisition and analysis can be found in the online Supporting Information.

### First and second level general linear model

Prior to DCM, a general linear model (GLM) was used to identify regions undergoing significant activation changes during task performance. The BOLD response at each voxel was convolved with the SPM12 canonical hemodynamic response function using a 128‐second high‐pass filter. Next, three contrasts of interest were defined for first‐level (single‐participant) analysis: (1) LookNegative ˃ LookNeutral, (2) Regulate ˃ LookNegative, and (3) LookNegative ˃ Regulate. The first contrast indexed brain activations associated with negative emotional reactivity, whereas the second and third contrasts indexed increases and decreases in activations during cognitive reappraisal. Contrast images for participants were then carried forward to a second‐level random‐effects GLM using a one‐sample *t* test design. For our GLM analyses, whole‐brain false discovery rate (FDR)‐corrected statistical thresholds were applied (*p*_FDR_ < 0.05), in addition to an arbitrary 10 voxel cluster‐extent threshold (KE ≥ 10 voxels).

### DCM

DCM uses a Bayesian framework to estimate how neural activity of one region (i.e., a node) influences activity in another region. As such, DCM provides more detailed and physiologically valid mapping between brain activity and psychological states than contrasts with correlation‐based functional connectivity, which is inherently undirected.

### Time series extraction

Following published guidelines [15], we determined the volumes of interest (VOIs) for the DCM model space informed by our obtained GLM results. Specifically, we extracted time series from three different prefrontal peaks, the right dlPFC, left dmPFC, and the left orbitofrontal cortex (OFC), which showed significant activation differences in the Regulate versus LookNegative contrast, and from a right amygdala cluster, which was significantly activated in the LookNegative > LookNeutral contrast. VOIs were defined as 4‐mm‐radius spheres centered on the group‐level (second‐level) analyses peak coordinates. Next, we identified the local maximum within these VOIs for each participant using a *p* < 0.05 uncorrected threshold. Participant‐level peaks were constrained to be a maximum of 8 mm away from the group‐level maximums. Using these criteria, from our sample of 102 adult women, VOI information was retrieved for 33 patients with obesity and 36 healthy‐weight control participants, which were included in our final DCM analysis.

### Model space specification

Candidate model spaces were specified using DCM 12.5 in SPM 12 (Statistical Parametric Mapping; version 7771; https://www.fil.ion.ucl.ac.uk/spm/software/spm12/). DCM models contain three components: endogenous connections between regions, also known as intrinsic parameters (DCM.A matrix), modulatory effects on these connections or extrinsic parameters by a task condition (DCM.B), and driving inputs (DCM.C) to regions themselves [16]. As described earlier, regions included in the model were the right amygdala, right dlPFC, left dmPFC, and left OFC. A full model was created for each participant (i.e., 4^2^ = 16 connectivity parameters), assuming bidirectional endogenous connections (DCM.A matrix) and bidirectional modulation by cognitive reappraisal (Regulate) between all regions. Driving input from emotional reactivity (LookNegative) was assumed for the right amygdala (DCM.C matrix).

### Parametric empirical Bayes model estimation

**FIGURE 2:**
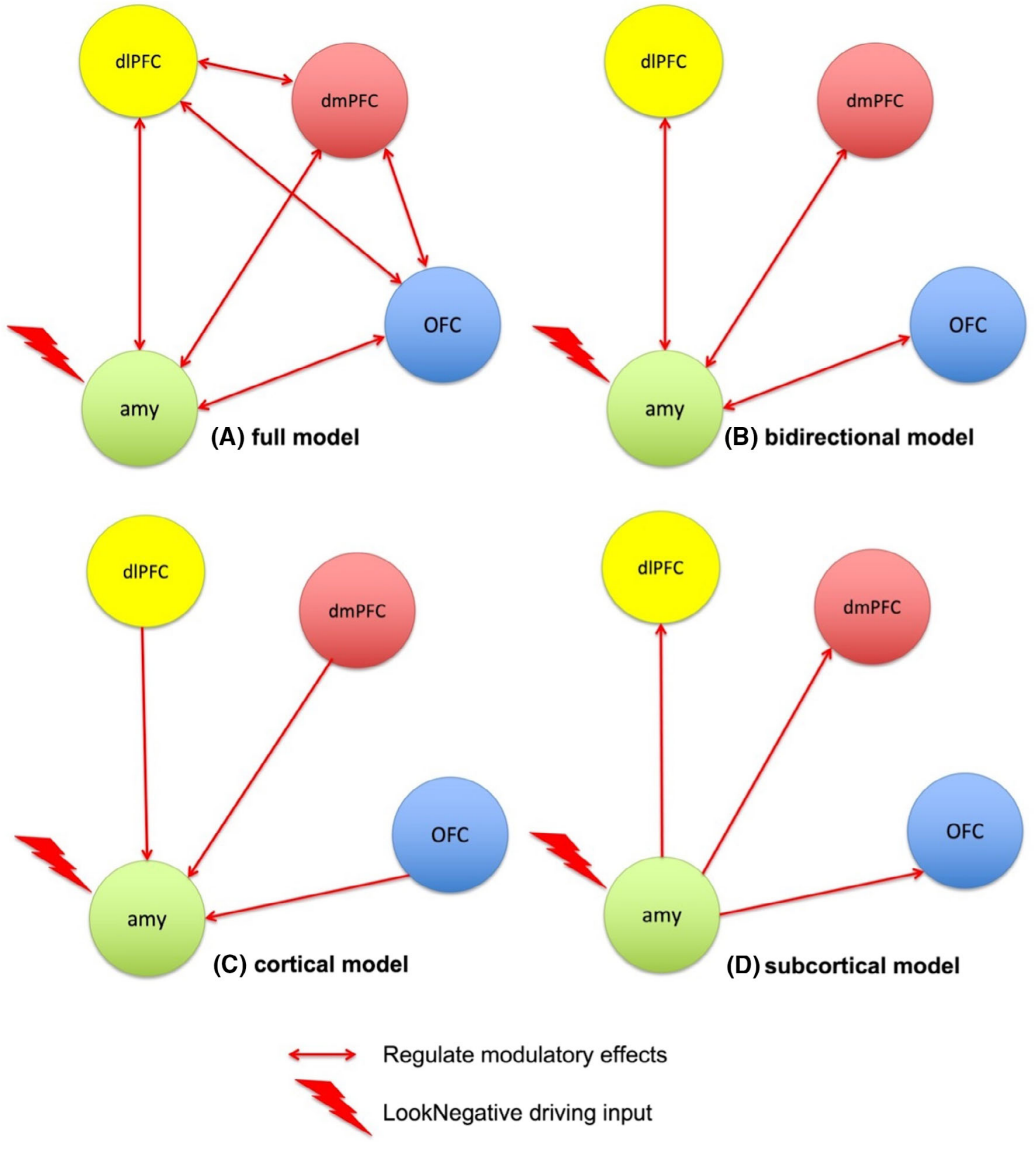
Candidate PEB models. Nested PEB models that differed on the location of the modulation by Regulate. The nodes making up the models were the right amygdala (green), the right dlPFC (yellow), the dmPFC (red), and the left OFC (blue). All models assumed bidirectional intrinsic connectivity between regions and a driving input by LookNegative into the amygdala. (A) Full model is fully modulated in all connections by Regulate. (B) Bidirectional model with Regulate modulating all connections from the amygdala to the dlPFC, dmPFC, and OFC, and vice versa. Cortical model (C) with connections from the dlPFC, dmPFC, and OFC to the amygdala modulated by Regulate and (D) where only connections from the amygdala to the dlPFC, dmPFC, and OFC were modulated by Regulate. dlPFC, dorsolateral prefrontal cortex; dmPFC, dorsomedial prefrontal cortex; OFC, orbitofrontal cortex; PEB, parametric empirical Bayes [Color figure can be viewed at wileyonlinelibrary.com]

After specification, using a parametric empirical Bayes (PEB) framework for DCM, all individual connectivity parameters of interest were tested at the group level to characterize how individual differences in neural circuitry were accounted for by BMI. Within the PEB framework, this is completed by iteratively comparing different reduced models. We defined five models, which differed on which pathways Regulate exerted a modulatory influence. These were based on predominant theoretical frameworks of emotional regulation, which describe a top‐down PFC appraisal system acting on the amygdala [17], including the following (Figure 2):

- A full model, with Regulate modulating all connections, including connections between prefrontal regions.
- A bidirectional model, with Regulate modulating all connections from the amygdala to the dlPFC, dmPFC, and OFC, and vice versa, while excluding connections between prefrontal regions.
- A cortical model in which connections from the dlPFC, dmPFC, and OFC to the amygdala were modulated by Regulate.
- A subcortical model in which connections from the amygdala to the dlPFC, dmPFC, and OFC were modulated by Regulate.
- And a null model without any fronto‐amygdalar modulation by Regulate.

Bayesian model comparison was used to compare the full PEB model against the nested PEB models. Last, we computed the Bayesian model average, the average of parameter values across models weighted by each model's posterior probability. The Bayesian model average was thresholded to retain only parameters with a posterior probability > 75% [18] to determine the optimal (winning) model from all candidate models.

### PEB: design matrix specification

The *spm.dcm.peb* function was used to specify and estimate the PEB model, which included the group‐level design matrix indicating the between‐participant parameters to be tested. This matrix included a first column including ones (to model across participant commonalities; that is, the constant or group mean) and BMI in the second column. In addition, we included age as a confounding regressor. All regressors were mean centered so that the intercept was interpretable as the mean connectivity value. This approach allowed us to obtain the mean connectivity strength across all participants and to determine how differences in connection strength were influenced by BMI.

Finally, to determine predictive validity (i.e., whether BMI can be predicted from the individual connections in the final reduced model), leave‐one‐out cross validation was performed [19]. This procedure iteratively fits the PEB model to all but one participant and predicts the covariate of interest BMI for the excluded participant.

## RESULTS

### Sociodemographic and clinical results

Sociodemographic information on the study sample is summarized in Table 1. As expected, participants with obesity had a significantly higher body fat percentage and BMI than control participants (*p* < 0.001). Participants with BMI > 30 were older than those with BMI < 30 (*p* < 0.001), and DERS scores were also significantly higher in the obesity group in comparison to lean participants (*p* < 0.001).

**TABLE 1:** Sample characteristics

Variable	HC (*n* = 36)		OB (*n* = 33)			
	Mean	SD	Mean	SD	*t*	*p*
Age (y)	31.64	10.54	41.61	9.63	4.08	<**0.001**
BMI	21.04	2.01	43.92	6.50	19.11	<**0.001**
Body fat percentage	14.02	2.23	55.48	12.83	17.64	<**0.001**
DERS	65.08	14.03	85.91	23.53	4.41	<**0.001**

*Note*: Bold indicates significant difference (*p* < 0.05).

Abbreviations: DERS, Difficulties in Emotion Regulation Scale; HC, healthy control; OB, obesity.

### fMRI task behavioral results

Participant in‐scanner ratings demonstrated that negative affect was significantly higher during the LookNegative condition (mean = 3.71 ± 0.91) in comparison to the LookNeutral condition (mean = 1.52 ± 0.76; *p* < 0.001), indicating significant emotional reactivity across participants. Importantly, negative affect was significantly reduced during the Regulate condition compared with the LookNegative condition, indicating successful cognitive reappraisal across participants (mean = 3.18 ± 1.08; *p* < 0.001).

### GLM

**FIGURE 3:**
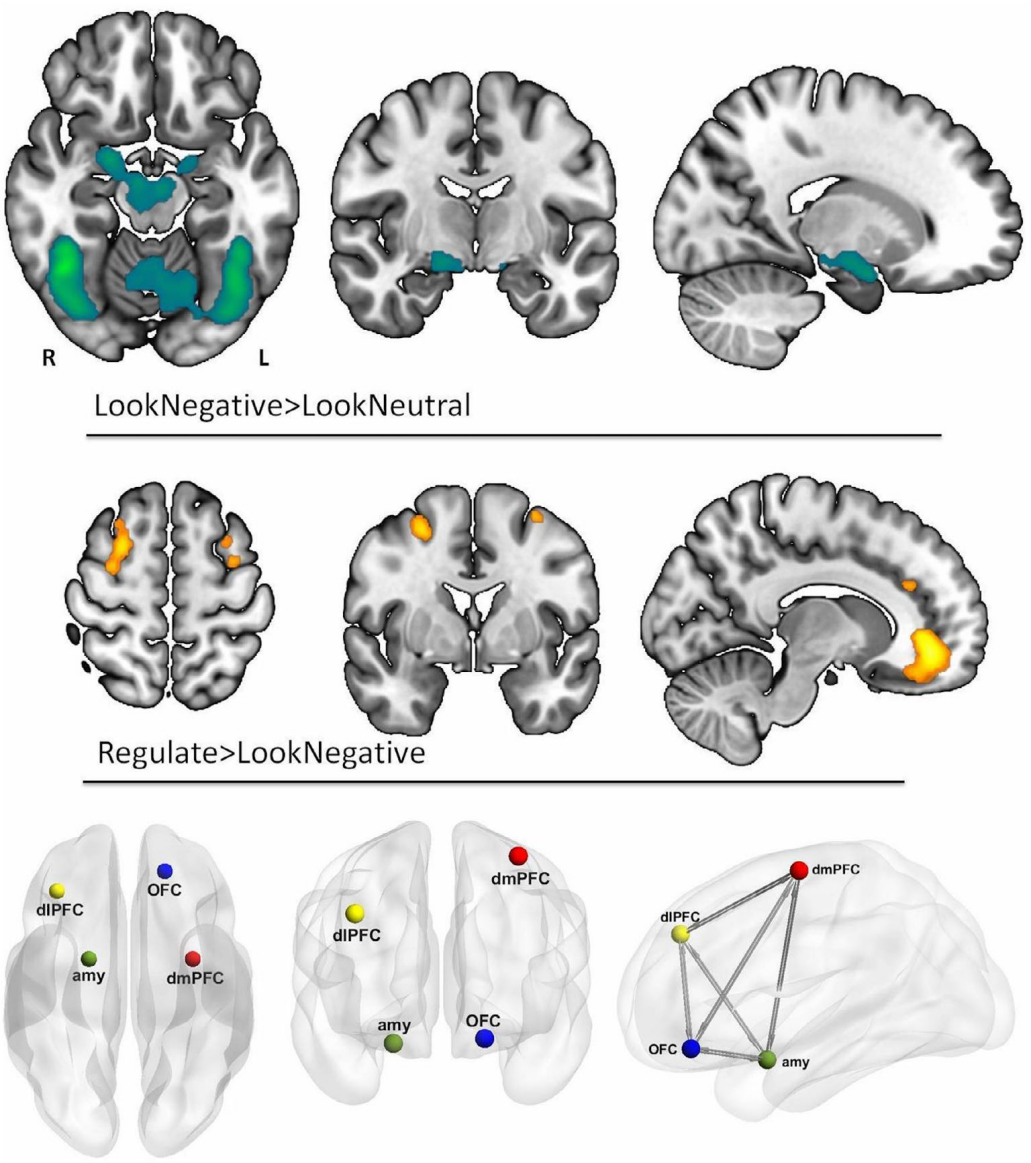
GLM results and DCM node placement. The first and second rows depict the GLM results of LookNegative vs. LookNeutral and Regulate vs. LookNegative contrasts, respectively, in both study groups, *p*_FDR_ < 0.05, 10 voxel cluster‐extent threshold (KE ≥ 10 voxels). The third row displays the nodes included for the DCM model space: the right amygdala (green), the right dlPFC (yellow), the left dmPFC (red), and the left OFC (blue). The last image is the representation of our model assuming bidirectional intrinsic connectivity between all DCM nodes. DCM, dynamic causal modeling; dlPFC, dorsolateral prefrontal cortex; dmPFC, dorsomedial prefrontal cortex; GLM, general linear model; OFC, orbitofrontal cortex [Color figure can be viewed at wileyonlinelibrary.com]

Akin to previous cognitive reappraisal studies [20, 21], we identified significant activations in the PFC during the Regulate > LookNegative contrast, whereas significant amygdala activations were observed in the LookNegative > LookNeutral contrast. We selected the peak maximums from these two contrasts to define our VOIs and extract the time series (right amygdala, right dlPFC, left dmPFC, and left OFC) for our DCM model space. These VOIs are depicted in Figure 3, and the Montreal Neurological Institute coordinates for these regions are presented in Table 2. See Supporting Information Tables S1 and S2 for the complete GLM results.

**TABLE 2:** Location of the GLM group maximums for VOIs included in the DCM model space

Region	MNI coordinates			t	Cluster KE
	x	y	z		
Right amygdala	20	−2	−18	10.58	904
Left dmPFC	−32	−2	60	3.88	42
Right dlPFC	36	32	36	4.88	1187
Left OFC	−18	42	−16	5.69	2043

*Note*: GLM results contrast images for both groups of participants. Three different prefrontal clusters, the right dlPFC, left dmPFC, and the OFC, which showed significant activation increases in the Regulate > LookNegative contrast and right amygdala cluster, which was significantly activated in the LookNegative > LookNeutral contrast. *p*_FDR_ < 0.05, 10 voxel cluster‐extent threshold (KE ≥ 10 voxels).

Abbreviations: DCM, dynamic causal modeling; dmPFC, dorsomedial prefrontal cortex; dlPFC, dorsolateral prefrontal cortex; GLM, general linear model; cluster KE, extent in voxels; MNI, Montreal Neurological Institute; OFC, orbitofrontal cortex; VOIs, volumes of interest.

### BMI‐associated differences in fronto‐amygdalar connectivity during cognitive reappraisal

**FIGURE 4:**
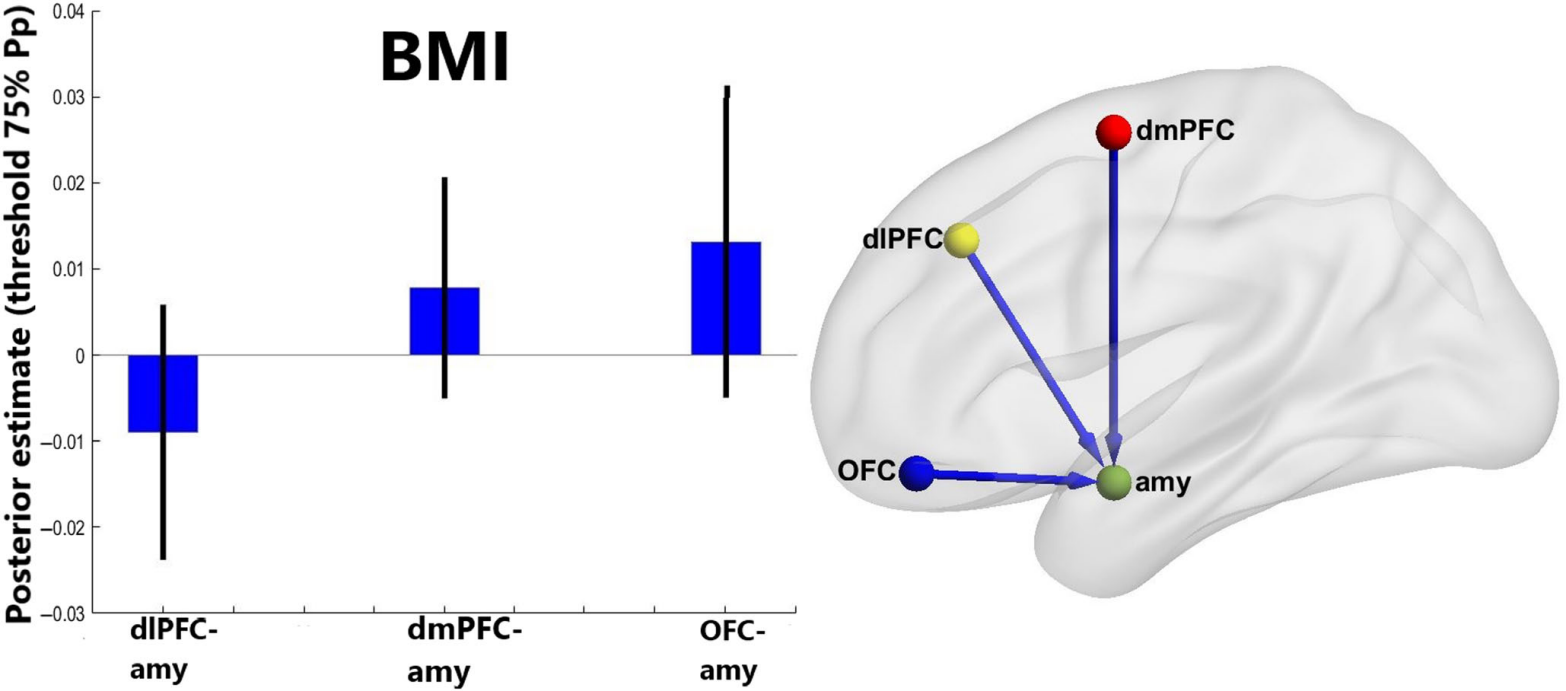
Second‐level dynamic causal modeling modulatory effects by emotional regulation. Average of the parameter Bayesian model average differences due to BMI. Differences due to BMI in the cortical model; in this model the connections from the three prefrontal clusters to the right amygdala are modulated by Regulate. Bar plots are thresholded for parameters >75% Pp and error bars correspond to 90% Bayesian CI, computed from the leading diagonal of the covariance matrix. Nodes displayed are: the right amygdala (green), the right dlPFC (yellow), the left dmPFC (red), and the left OFC (blue). dlPFC, dorsolateral prefrontal cortex; dmPFC, dorsomedial prefrontal cortex; Pp, posterior probability; OFC, orbitofrontal cortex [Color figure can be viewed at wileyonlinelibrary.com]

**FIGURE 5:**
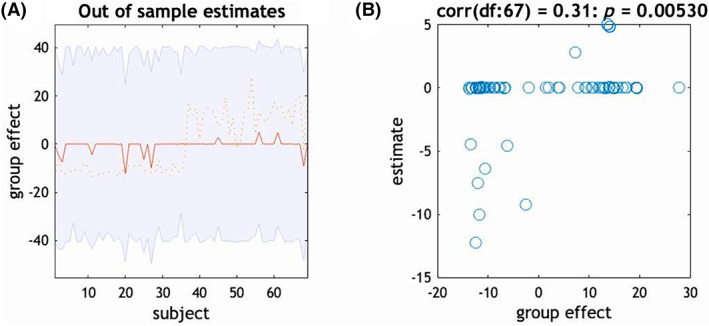
(A) Leave‐one‐out cross validation to the modulation from the OFC to the amygdala. The out‐of‐samples estimate of BMI scores for each participant (red line) with 90% CI (shaded area). The dashed orange line is the actual group effect. (B) Participant BMI scores can be reliably predicted based on their modulation of OFC‐to‐amygdala effective connectivity during emotional regulation (*p* = 0.005, *r* = 0.31). OFC, orbitofrontal cortex [Color figure can be viewed at wileyonlinelibrary.com]

The cortical model best explained the differences in fronto‐amygdalar modulation during cognitive reappraisal due to BMI with a posterior probability of 86%. Significant model parameters are presented in Table 3. As shown in Figure 4, participants with a higher BMI were less effective at inhibiting activity in the amygdala via the OFC and dmPFC during cognitive reappraisal compared with those with a lower BMI. Leave‐one‐out cross validation revealed that the modulatory effect of cognitive reappraisal on OFC‐to‐amygdala connectivity had the capacity to predict higher BMI (Figure 5; *r*(67) = 0.31, *p* < 0.005). In contrast, increased excitatory modulation of dlPFC‐to‐amygdala connectivity was found in participants with a lower BMI. We have included the results of an analysis featuring an age‐matched sample in Supporting Information Tables S3 and S4.

**TABLE 3:** DCM parameter estimates

DCM parameter	Model parameter	Effect size in Hz [90% CI]	Posterior probability
Modulation by emotional regulation [B]	BMI		
	dlPFC‐amy	−0.009 [0 to −0.02]	0.80
	dmPFC‐amy	0.008 [0 to 0.02]	0.80
	OFC‐amy	0.013 [0 to 0.03]	0.80

*Note*: Modulatory parameters represent context‐dependent changes in coupling between regions induced by emotional regulation.

Abbreviations: amy, amygdala; DCM, dynamic causal modeling; dlPFC, dorsolateral prefrontal cortex; dmPFC, dorsomedial prefrontal cortex; OFC, orbitofrontal cortex.

## DISCUSSION

This study used DCM to determine how BMI impacts fronto‐amygdalar effective connectivity during cognitive reappraisal. We found strong evidence to support that higher BMI is associated with less effective inhibitory modulation of the amygdala by prefrontal control regions during reappraisal. Specifically, our neuroimaging time series data were best explained by a model configuration in which higher BMI was linked to a causal effective connectivity architecture defined by reduced downregulation of amygdala activity by the OFC and the dmPFC. Moreover, we found that the dlPFC had stronger modulatory effects on the amygdala in patients with a lower BMI.

Circuit models of emotional regulation have posited that the OFC acts as a primary prefrontal conduit to modulate activity in subcortical regions [22], which is facilitated by its dense reciprocal connections with the amygdala via the uncinate fasciculus [23]. The OFC is implicated in inhibitory control, and recent studies have highlighted how OFC function is impacted in individuals with higher BMI [24]. Serving as a key center for allocating value to rewards and orchestrating motivated eating behavior [25], our evidence of decreased OFC modulation of amygdala activity during effortful emotional regulation aligns with predominant models of emotion‐regulation impairment, and it is indicative of dysfunction in this pathway potentially contributing to deficits in both affect regulation and food‐related inhibitory control [26]. In addition, leave‐one‐out cross validation analysis on OFC‐amygdala effective connectivity parameters demonstrated that BMI values could be reliably predicted by the extent to which participants were able to modulate this pathway. As previous research has identified decreased OFC gray matter density [27] and total OFC volume [28] in participants with higher BMI, altered OFC function could represent a distinguishing feature of higher BMI and a mechanism through which the reinforcing properties of food are used to alleviate negative affect. This is supported by recent translational research identifying a glutamatergic OFC‐lateral hypothalamus circuit as a novel stress‐sensitive anorexigenic neural pathway involved in the cortical control of food intake [29].

Parallels between our OFC‐amygdala finding can be made with our evidence of altered dmPFC function during affective processing [30]. Alterations in both pathways have been found to contribute to deficits in mentalizing (i.e., formulating thoughts surrounding internal mental states and intentions) [31], although the dmPFC is understood to be specifically involved in volitional emotional regulation as neurofeedback training has been shown to lead to an increase in top‐down connectivity from the dmPFC onto the amygdala [32]. Decreases in dmPFC activity during emotional regulation have been described in other psychiatric disorders that are often highly comorbid with obesity [33], including mood and anxiety disorders, [34], depression [35], and generalized anxiety disorder [36].

Our study complements previous research demonstrating that changes in effective connectivity between dorsal and ventral prefrontal regions moderate emotional regulation [37], as we found that dlPFC‐to‐amygdala modulation during cognitive reappraisal was associated with lower BMI. dlPFC‐mediated processes, such as working memory, are essential to the successful execution of cognitive reappraisal given that model‐based control techniques (i.e., the manipulating and updating of appraisals) is underpinned by this circuitry [38]. In the context of food intake, dlPFC activity directly after a diet has been found to predict real‐world diet success in patients with obesity at a 1‐year follow‐up, suggesting that impaired dlPFC function may underlie risk factors such as ineffective affect regulation and impulse control [39]. Likewise, dlPFC response during food cue presentation, in combination with GLP‐1 levels, has been found to individually forecast subsequent weight change. However, it is worth noting that anatomical studies have shown that the dlPFC is sparsely connected to the amygdala and that it has minimal projections from the amygdala [22]. As such, it is likely that the impact of the dlPFC on the amygdala is via an indirect pathway or pathways [40], which may contribute to the disrupted balance between goal‐directed and habitual control systems and between internal/external monitoring processes found in populations at higher weight [41].

There are several limitations to the present study. Our sample consisted only of women, and it would be beneficial to feature more diverse samples moving forward. In relation to our DCM analyses, this hypothesis‐driven technique relies on a priori assumptions on which brain regions to include in a model space and is therefore limited in the number of regions that can be evaluated. In this sense, the dual opposing system mechanism mentioned previously does not fully capture the nuances of recent findings suggesting the involvement of thalamic and subcortical hubs during affect processing and model‐based cognition [42] or include subcortical regions proposed to contribute to overeating [43]. For instance, alterations in insula‐prefrontal connectivity have been associated with emotion‐regulation deficits in individuals at high weight and could be investigated using DCM in the future [6]. Moreover, although our fronto‐amygdala network aligns with previous models of emotional regulation, mapping a system including other regions that are modulated by cognitive reappraisal would be of interest. Using a causal search algorithm (e.g., GIMME), which is capable of including more regions than DCM, could support more complex model explanations of cognitive reappraisal in individuals at higher weight. It is also worth noting that the cross‐sectional nature of our study design does not allow for inferences to be made regarding causality. Past research has linked child emotional reactivity to emotional and external eating [44], though it would be of interest to ascertain whether there are neurobiological markers for detecting increased risk before harmful eating behaviors ensue. Last, successful emotional regulation can be accomplished in many ways and here we examined only reappraisal. Future work should examine the neurobiological substrates of varied emotion‐regulation strategies (e.g., detachment, focusing on positive aspects, acceptance) to better understand how strategy selection impacts regulation success as a function of BMI, as well as examine the level of mental effort participants require while engaging in emotional regulation during scanning.

## CONCLUSION

This study represents a meaningful step forward in improving our understanding of how emotion‐regulation mechanisms might be affected in women with higher BMI. Using DCM, we mapped the directionality of impacted fronto‐amygdalar pathways during cognitive reappraisal and used a leave‐one‐out cross validation to pinpoint which circuitry can robustly identify higher BMI. Our findings align with and further uphold prevalent models of emotional regulation and support the notion that altered neurobiological function contributes to the difficulties in adequately assessing and managing negative affective states at higher weight.

## AUTHOR CONTRIBUTIONS

Pablo Maturana‐Quijada, Trevor Steward, Carles Soriano‐Mas, and Fernando Fernandez‐Aranda were involved in developing the research aims. Pablo Maturana‐Quijada, Trevor Steward, Holly J. Carey, and Carles Soriano‐Mas conducted the neuroimaging analysis and contributed to interpretation of the results. Trevor Steward, Nuria Vilarrasa, Fernando Guerrero‐Perez, Isabel Sánchez, Nuria Custal, Nuria Virgili, Rafael Lopez‐Urdiales, and Romina Miranda‐Olivos contributed to the data collection. Nuria Vilarrasa, Fernando Guerrero‐Perez, Isabel Sánchez, Nuria Custal, Nuria Virgili, and Rafael Lopez‐Urdiales contributed to the clinical and phenotypical characterization of participants. Pablo Maturana‐Quijada, Trevor Steward, Holly J. Carey, Carles Soriano‐Mas, Nuria Virgili, Susana Jiménez‐Murcia, José‐Antonio Fernández‐Formoso, Fernando Guerrero‐Perez, Romina Miranda‐Olivos, Isabel Sánchez, Nuria Custal, Nuria Virgili, Rafael Lopez‐Urdiales, and Fernando Fernandez‐Aranda were involved in writing and proofreading the manuscript. All authors have read and approved the final manuscript.

## FUNDING INFORMATION

We thank CERCA Program/Generalitat de Catalunya for institutional support. This manuscript and research were supported by a grant from the Instituto de Salud Carlos III (ISCIII) (FIS PI13/01958, PI14/00290, PI17/01167, PI19/01171, PI20/0132) and co‐funded by FEDER funds/European Regional Development Fund (ERDF), a way to build Europe. Trevor Steward is supported by a National Health and Medical Research Council (NHMRC)/Medical Research Future Fund (MRFF) Investigator Grant (MRF1193736), a Brain & Behavior Research Foundation (BBRF) Young Investigator Grant, and a University of Melbourne McKenzie Fellowship. Additional support was received from the EU Eat2beNice Grant (H2020‐SFS‐2016‐2; Ref 728018) and PRIME (PRIME 847879). CIBERobn, CIBERDEM, and CIBERsam are initiatives of ISCIII.

## CONFLICT OF INTEREST STATEMENT

Fernando Fernandez‐Aranda received consultancy honoraria from Novo Nordisk A/S and editorial honoraria as Editor in Chief from Wiley. The rest of the authors declare no conflict of interest. The funders had no role in the design of the study; in the collection, analysis, or interpretation of data; in the writing of the manuscript; or in the decision to publish the results.

## Supporting information


**Data S1.** Supporting information.

## References

[1] Spiegelman BM , Flier JS . Obesity and the regulation of energy balance. Cell. 2001;104:531‐543. 11239410 10.1016/s0092-8674(01)00240-9

[2] Giel KE , Bulik CM , Fernandez‐Aranda F , et al. Binge eating disorder. Nat Rev Dis Primers. 2022;8:16. doi:10.1038/s41572-022-00344-y 35301358 PMC9793802

[3] Gross JJ , Thompson R . Emotion regulation. In: Gross JJ , ed. Conceptual Foundations. Handbook of Emotion Regulation. The Guilford Press; 2007:3‐27.

[4] Steward T , Davey CG , Jamieson AJ , et al. Dynamic neural interactions supporting the cognitive reappraisal of emotion. Cereb Cortex. 2020;31:961‐973. 10.1093/cercor/bhaa268 32960214

[5] Buhle JT , Silvers JA , Wage TD , et al. Cognitive reappraisal of emotion: a meta‐analysis of human neuroimaging studies. Cereb Cortex. 2014;24:2981‐2990. 23765157 10.1093/cercor/bht154 PMC4193464

[6] Steward T , Picó‐Pérez M , Mata F , et al. Emotion regulation and excess weight: impaired affective processing characterized by dysfunctional insula activation and connectivity. PLoS One. 2016;11:e0152150. doi:10.1371/journal.pone.0152150 27003840 PMC4803189

[7] Steward T , Picó‐Pérez M , Mestre‐Bach G , et al. A multimodal MRI study of the neural mechanisms of emotion regulation impairment in women with obesity. Transl Psychiatry. 2019;9:194. doi:10.1038/s41398-019-0533-3 31431608 PMC6702163

[8] Friston KJ , Harrison L , Penny W . Dynamic causal modelling. Neuroimage. 2003;19:1273‐1302. 12948688 10.1016/s1053-8119(03)00202-7

[9] Sheehan DV , Lecrubier Y , Sheehan KH , et al. The Mini‐International Neuropsychiatric Interview (M.I.N.I): the development and validation of a structured diagnostic psychiatric interview for DSM‐IV and ICD‐10. J Clin Psychiatry. 1998;59(suppl 20):22‐33. 9881538

[10] Gratz KL , Roemer L . Multidimensional assessment of emotion regulation and dysregulation: development, factor structure, and initial validation of the difficulties in Emotion Regulation Scale. J Psychopathol Behav Assess. 2004;26:41‐54.

[11] Browning LM , Mugridge O , Dixon AK , Aitken SW , Prentice AM , Jebb SA . Measuring abdominal adipose tissue: comparison of simpler methods with MRI. Obes Facts. 2011;4:9‐15. 21372606 10.1159/000324546 PMC6450044

[12] Phan KL , Fitzgerald DA , Nathan PJ , Moore GJ , Uhde TW , Tancer ME . Neural substrates for voluntary suppression of negative affect: a functional magnetic resonance imaging study. Biol Psychiatry. 2005;57:210‐219. 15691521 10.1016/j.biopsych.2004.10.030

[13] Patel AX , Kundu P , Rubinov M , et al. A wavelet method for modeling and despiking motion artifacts from resting‐state fMRI time series. Neuroimage. 2014;95:287‐304. 24657353 10.1016/j.neuroimage.2014.03.012 PMC4068300

[14] Whitfield‐Gabrieli S , Nieto‐Castanon A . Conn: a functional connectivity toolbox for correlated and anticorrelated brain networks. Brain Connect. 2012;2:125‐141. 22642651 10.1089/brain.2012.0073

[15] Zeidman P , Jafarian A , Corbin N , et al. A guide to group effective connectivity analysis, part 1: first level analysis with DCM for fMRI. Neuroimage. 2019;200:174‐190. 31226497 10.1016/j.neuroimage.2019.06.031 PMC6711459

[16] Stephan KE , Weiskopf N , Drysdale PM , Robinson PA , Friston KJ . Comparing hemodynamic models with DCM. Neuroimage. 2007;38:387‐401. 17884583 10.1016/j.neuroimage.2007.07.040 PMC2636182

[17] Ochsner KN , Silvers JA , Buhle JT . Functional imaging studies of emotion regulation: a synthetic review and evolving model of the cognitive control of emotion. Ann N Y Acad Sci. 2012;1251:E1‐E24. 23025352 10.1111/j.1749-6632.2012.06751.x PMC4133790

[18] Kass RE , Raftery AE . Bayes factors. J Am Stat Assoc. 1995;90:773‐795.

[19] Zeidman P , Jafarian A , Seghier ML , et al. A guide to group effective connectivity analysis, part 2: second level analysis with PEB. Neuroimage. 2019;200:12‐25. 31226492 10.1016/j.neuroimage.2019.06.032 PMC6711451

[20] Stephanou K , Davey CG , Kerestes R , et al. Brain functional correlates of emotion regulation across adolescence and young adulthood. Hum Brain Mapp. 2016;37:7‐19. 26596970 10.1002/hbm.22905 PMC6867496

[21] Stephanou K , Davey CG , Kerestes R , Whittle S , Harrison BJ . Hard to look on the bright side: neural correlates of impaired emotion regulation in depressed youth. Soc Cogn Affect Neurosci. 2017;12:1138‐1148. 28402574 10.1093/scan/nsx039 PMC5490679

[22] Ray RD , Zald DH . Anatomical insights into the interaction of emotion and cognition in the prefrontal cortex. Neurosci Biobehav Rev. 2012;36:479‐501. 21889953 10.1016/j.neubiorev.2011.08.005 PMC3244208

[23] Bhatia K , Henderson L , Yim M , Hsu E , Dhaliwal R . Diffusion tensor imaging investigation of uncinate fasciculus anatomy in healthy controls: description of a subgenual stem. Neuropsychobiology. 2018;75:132‐140. 10.1159/000485111 29332063

[24] Parsons N , Steward T , Clohesy R , Almgren H , Duehlmeyer L . A systematic review of resting‐state functional connectivity in obesity: refining current neurobiological frameworks and methodological considerations moving forward. Rev Endocr Metab Disord. 2021;23(4):861‐879. 34159504 10.1007/s11154-021-09665-x

[25] Seabrook LT , Borgland SL . The orbitofrontal cortex, food intake and obesity. J Psychiatry Neurosci. 2020;45:304‐312. 32167268 10.1503/jpn.190163 PMC7850155

[26] Steward T , Miranda‐Olivos R , Soriano‐Mas C , Fernández‐Aranda F . Neuroendocrinological mechanisms underlying impulsive and compulsive behaviors in obesity: a narrative review of fMRI studies. Rev Endocr Metab Disord. 2019;20(3):263‐272. 31654260 10.1007/s11154-019-09515-x

[27] Walther K , Birdsill AC , Glisky EL , Ryan L . Structural brain differences and cognitive functioning related to body mass index in older females. Hum Brain Mapp. 2010;31:1052‐1064. 19998366 10.1002/hbm.20916 PMC6870943

[28] Cazettes F , Cohen JI , Yau PL , Talbot H , Convit A . Obesity‐mediated inflammation may damage the brain circuit that regulates food intake. Brain Res. 2011;1373:101‐109. 21146506 10.1016/j.brainres.2010.12.008 PMC3026911

[29] Clarke RE , Voigt K , Reichenbach A , et al. Identification of a stress‐sensitive anorexigenic neurocircuit from medial prefrontal cortex to lateral hypothalamus. Biol Psychiatry. 2022;93(4):309‐321. 36400605 10.1016/j.biopsych.2022.08.022

[30] Dörfel D , Lamke JP , Hummel F , Wagner U , Erk S , Walter H . Common and differential neural networks of emotion regulation by detachment, reinterpretation, distraction, and expressive suppression: a comparative fMRI investigation. Neuroimage. 2014;101:298‐309. 24993897 10.1016/j.neuroimage.2014.06.051

[31] Dixon ML , Thiruchselvam R , Todd R , Christoff K . Emotion and the prefrontal cortex: an integrative review. Psychol Bull. 2017;143:1033‐1081. 28616997 10.1037/bul0000096

[32] Koush Y , Meskaldji DE , Pichon S , et al. Learning control over emotion networks through connectivity‐based neurofeedback. Cereb Cortex. 2017;27:1193‐1202. 26679192 10.1093/cercor/bhv311

[33] Simon GE , von Korff M , Saunders K , et al. Association between obesity and psychiatric disorders in the us adult population. Arch Gen Psychiatry. 2006;63:824‐830. 16818872 10.1001/archpsyc.63.7.824 PMC1913935

[34] Picó‐Pérez M , Radua J , Steward T , Menchón JM , Soriano‐Mas C . Emotion regulation in mood and anxiety disorders: a meta‐analysis of fMRI cognitive reappraisal studies. Prog Neuropsychopharmacol Biol Psychiatry. 2017;79(Pt B):96‐104. 28579400 10.1016/j.pnpbp.2017.06.001

[35] la Peña‐Arteaga VD , Berruga‐Sánchez M , Steward T , et al. An fMRI study of cognitive reappraisal in major depressive disorder and borderline personality disorder. Eur Psychiatry. 2021;64:e56. doi:10.1192/j.eurpsy.2021.2231 34465401 PMC8516744

[36] Manber Ball T , Ramsawh HJ , Campbell‐Sills L , Paulus MP , Stein MB . Prefrontal dysfunction during emotion regulation in generalized anxiety and panic disorders. Psychol Med. 2013;43:1475‐1486. 23111120 10.1017/S0033291712002383 PMC4308620

[37] Morawetz C , Bode S , Baudewig J , Kirilina E , Heekeren HR . Changes in effective connectivity between dorsal and ventral prefrontal regions moderate emotion regulation. Cereb Cortex. 2016;26:1923‐1937. 25631055 10.1093/cercor/bhv005

[38] Etkin A , Büchel C , Gross JJ . The neural bases of emotion regulation. Nat Rev Neurosci. 2015;16(11):693‐700. 26481098 10.1038/nrn4044

[39] Weygandt M , Mai K , Dommes E , et al. Impulse control in the dorsolateral prefrontal cortex counteracts post‐diet weight regain in obesity. Neuroimage. 2015;109:318‐327. 25576647 10.1016/j.neuroimage.2014.12.073

[40] Mcdonald AJ . Cortical pathways to the mammalian amygdala. Prog Neurobiol. 1998;55:257‐332. 9643556 10.1016/s0301-0082(98)00003-3

[41] Legget KT , Wylie KP , Cornier MA , Berman BD , Tregellas JR . Altered between‐network connectivity in individuals prone to obesity. Physiol Behav. 2021;229:113242. doi:10.1016/j.physbeh.2020.113242 33157075 PMC7775284

[42] Steward T , Kung PH , Davey CG , et al. A thalamo‐centric neural signature for restructuring negative self‐beliefs. Mol Psychiatry. 2021;27(3):1611‐1617. 10.1038/s41380-021-01402-9 PMC9095461 34974523

[43] Kung PH , Soriano‐Mas C , Steward T . The influence of the subcortex and brain stem on overeating: how advances in functional neuroimaging can be applied to expand neurobiological models to beyond the cortex. Rev Endocr Metab Disord. 2022;23:719‐731. 35380355 10.1007/s11154-022-09720-1 PMC9307542

[44] Harrist AW , Hubbs‐Tait L , Topham GL , Shriver LH , Page MC . Emotion regulation is related to children's emotional and external eating. J Dev Behav Pediatr. 2013;34:557‐565. 24131878 10.1097/DBP.0b013e3182a5095f

