# Supplementary material for: Dynamic fronto‐amygdalar interactions underlying emotion‐regulation deficits in women at higher weight
Source: Obesity (Silver Spring). 2023 Aug 6;31(9):2283–93. doi: 10.1002/oby.23830 (PMC10946850; doi:10.1002/oby.23830)
Supplement: Supplementary file 1 — Data S1. Supporting information. [file OBY-31-2283-s001.docx]

Supplementary Information

**Dynamic frontoamygdalar interactions underlying emotion regulation deficits in women at higher weight**

Pablo Maturana-Quijada^a,†^, Trevor Steward^b,c†^, Nuria Vilarrasa^d,e^, Romina Miranda-Olivos^f,h^, Susana Jiménez-Murcia^f,g,h,i^, Holly J. Carey ^c^, José-Antonio Fernández-Formoso^j^, Fernando Guerrero-Perez^d^, Isabel Sánchez^f,h,i^, Nuria Custal^i^, Nuria Virgili^d,e^, Rafael Lopez-Urdiales^d^, Carles Soriano-Mas^a,j,k^, Fernando Fernandez-Aranda.^,f,g,h,i^

^a^ Psychiatry and Mental Health Group, Neuroscience Program, Institut d’ Investigació Biomèdica de Bellvitge – IDIBELL, L’ Hospitalet de Llobregat (Barcelona), Spain.

^b^ Melbourne School of Psychological Sciences, Faculty of Medicine, Dentistry and Health Sciences, University of Melbourne, Parkville, Victoria, Australia.

^c^ Melbourne Neuropsychiatry Centre, Department of Psychiatry, Faculty of Medicine, Dentistry and Health Sciences, University of Melbourne, Parkville, Victoria, Australia.

^d^ Department of Endocrinology and Nutrition, University Hospital of Bellvitge-IDIBELL, C/Feixa Llarga s/n, 08907, Barcelona, Spain

^e^ CIBER Diabetes and Associated Metabolic Diseases (CIBERDEM), Instituto de Salud Carlos III, Barcelona, Spain

^f^ Ciber Fisiopatología Obesidad y Nutrición (CIBERObn), Instituto Salud Carlos III, Barcelona, Spain

^g^ Department of Clinical Sciences, School of Medicine and Health Sciences, University of Barcelona, Spain

^h^ Psychoneurobiology of Eating and Addictive Behaviors Group, Neurosciences Program, Bellvitge Biomedical Research Institute (IDIBELL), 08908 Barcelona, Spain

^i^ Clinical Psychology Unit, University Hospital of Bellvitge, 08907 Hospitalet del Llobregat, Spain

^j^ Ciber Salud Mental (CIBERSAM), Instituto Salud Carlos III, Barcelona, Spain.

^k^ Department of Social Psychology and Quantitative Psychology, School of Psychology, University of Barcelona, Spain

*^†^Pablo Maturana-Quijada and Trevor Steward share first authorship.*

**This file includes:**

Supplementary text

Table S1

Table S2

Table S3

Table S4

**MRI image acquisition**

Participants were scanned using a 3T Phillips Ingenia system (Philips Medical Systems) equipped with a thirty-two-channel phased-array head coil. Two functional MRI (fMRI) sequences (i.e., one during resting-state and another during the completion of the emotion regulation task, see below) were acquired with identical parameters: a single-shot gradient-echo echo-planar imaging (EPI) was used. This sequence feature: repetition time, 2000 msec; echo time, 25 msec; and pulse angle, 90°; in a 24-cm field of view; and an 80 × 80-pixel matrix; providing isotropic voxel sizes of 3 × 3 x 3 mm, with no gap. 40 interleaved sections, parallel to the anterior-posterior commissure line, were acquired for each whole-brain volume.

A high-resolution T1-weighted anatomical scan was also acquired. We used a three-dimensional fast-spoiled gradient, inversion-recovery sequence with 233 contiguous slices (repetition time, 10.43 msec; echo time, 4.8 msec; flip angle, 8°) in a 24-cm field of view, with a 320 × 320 pixel matrix and isotropic voxel sizes of 0.75 × 0.75 x 0.75 mm.

**Emotion regulation task**

Prior to scanning, participants were given instruction on how to utilize reappraisal strategies. Three types of reinterpretations were recommended to participants using an example situation: (1) the scene is not real (e.g. the people on the screen are actors); (2) the situation will likely get better with time; and (3) the situation is not as grave as it first appears (e.g. seeing the situation in a more positive light). Participant were specifically instructed that they were not to use non-cognitive strategies (i.e. as looking away) during stimulus presentation.

Each block began with the instructive prompt (LookNeutral, LookNegative or Regulate) presented in the middle of the screen for four seconds. After the prompt, participants viewed two different pictures of equal valence for ten seconds each. After the presentation of the second picture of each block, the intensity of the participants’ distress was self-rated on a 1–5 numeric scale (1 being ‘neutral’ and 5 being ‘extremely negative’) in order to confirm whether participants were successfully carrying out the task instructions. Interactions between in-scanner ratings for each condition (LookNeutral, LookNegative or Regulate) and subject groups were evaluated using a 2x3 repeated-measures ANOVA analysis.

24 images from the International Affective Picture System [1] were used for the task: eight neutral pictures (e.g. household objects), which were presented in the LookNeutral condition, and 16 highly unpleasant pictures (e.g. mutilations) in the LookNegative and Regulate conditions. In total, the task consisted of twelve, 20-second blocks including the presentation of two images for ten seconds, with a total of four blocks for each condition. Instructions (LookNeutral, LookNegative or Regulate) were pseudo-randomized throughout the task to avoid the induction of sustained mood states. In-scanner ratings were recorded through an fMRI-compatible response pad (Lumina–Cedrus Corporation). Each block was followed by a 10-second presentation of a cross fixation to separate each block and to minimize carry-over effects. Task instructions and visual stimuli were presented using Presentation® software (Version 18.3, build 03.11.16, www. neurobs.com) through an MRI-compatible BOLD screen (BOLD screen 32, Cambridge Research Systems) located at the rear of the scanner gantry. An angled mirror system was used to allow participants to view the images.

Table S1. Activations for the Cognitive Reappraisal Task

| Contrast | Cluster size | $p_{FDR}$ | Peak t-value | x | y | z | Brain region |
| --- | --- | --- | --- | --- | --- | --- | --- |
| Regulate>LookNegative | 2043 | 0.004 | 5.69 | -18 | 42 | -16 | Frontal_Sup_Orb_L |
|  |  |  |  | 18 | 40 | -18 | Caudate_R |
|  |  |  |  | 20 | 48 | -10 | Frontal_Orb_R |
|  | 1187 | 0.006 | 4.88 | 36 | 32 | 36 | Frontal_Mid_R |
|  |  |  |  | 26 | 18 | 48 | Frontal_Sup_R |
|  |  |  |  | 48 | 16 | 40 | Frontal_Mid_R |
|  | 260 | 0.007 | 4.55 | -58 | -54 | 28 | SupraMarginal_L |
|  | 646 | 0.007 | 4.51 | 60 | -52 | 28 | Angular_R |
|  |  |  |  | 56 | -50 | 36 | Angular_R |
|  |  |  |  | 48 | -48 | 32 | Angular_R |
|  | 111 | 0.012 | 4.01 | -46 | -44 | 50 | Parietal_Inf_L |
|  | 42 | 0.343 | 3.88 | -32 | -2 | 60 | Frontal_Mid_R |
|  |  |  |  | -28 | 8 | 58 | Frontal_Mid_L |
|  | 64 | 0.015 | 3.86 | 8 | 34 | 26 | Cingulum_Ant_R |
|  | 67 | 0.017 | 3.78 | 68 | -32 | -6 | Temporal_Mid_R |
|  | 17 | 0.023 | 3.60 | 50 | 32 | -16 | Frontal_Inf_Orb_R |
|  | 19 | 0.028 | 3.50 | -42 | 14 | 48 | Frontal_Mid_L |
|  |  |  |  | -44 | 6 | 50 | Precentral_L |
|  | 26 | 0.028 | 3.50 | 52 | -28 | 44 | Postcentral_R |
|  | 30 | 0.030 | 3.46 | -62 | -34 | 34 | SupraMarginal_L |
|  | 22 | 0.033 | 3.41 | 38 | 42 | -6 | Pars_Orb_R |
|  | 11 | 0.034 | 3.30 | -54 | -44 | -4 | Temporal_Mid_L |
|  | 28 | 0.034 | 3.39 | 8 | -54 | 50 | Precuneus_R |
| LookNegative>Regulate | 13031 | 0.000 | 8.42 | 44 | -68 | -2 | Temporal_Mid_R |
|  |  |  |  | 40 | -46 | -22 | Fusiform_R |
|  |  |  |  | -6 | -90 | 18 | Cuneus_L |
|  | 270 | 0.001 | 4.65 | 0 | 66 | -12 | Frontal_Med_Orb_L |
|  | 272 | 0.002 | 4.24 | 0 | -56 | -46 | Cerebelum_L |
|  | 55 | 0.009 | 3.68 | -18 | -46 | -50 | Cerebelum_L |
|  | 124 | 0.014 | 3.49 | -42 | 22 | -20 | Temporal_Pole_Sup_L |
|  |  |  |  | -56 | 10 | -10 | Temporal_Pole_Sup_L |
|  | 129 | 0.018 | 3.37 | -48 | -8 | 14 | Rolandic_Oper_L |
|  |  |  |  | -36 | -14 | 20 | Insula_L |
|  | 32 | 0.019 | 3.34 | 28 | -78 | -36 | Cerebelum_Crus1_R |
|  | 34 | 0.024 | 3.23 | 18 | -54 | -54 | Cerebelum_R |
|  | 18 | 0.026 | 3.17 | -30 | -56 | -4 | Fusiform_L |
|  | 11 | 0.038 | 2.98 | 2 | -78 | -16 | Vermis |
| LookNegative>LookNeutral | 9701 | 0.000 | 17 | 44 | -68 | -4 | Temporal_Inf_R |
|  |  |  |  | 42 | -52 | -18 | Fusiform_R |
|  |  |  |  | -40 | -48 | -22 | Fusiform_L |
|  | 904 | 0.000 | 10.58 | 20 | -2 | -18 | Amygdala_R |
|  |  |  |  | -12 | -26 | -12 | S Nigra L |
|  |  |  |  | 8 | -14 | -16 | Ventral_Diencephalon_R |
|  | 115 | 0.000 |  | 0 | -84 | 16 | Cuneus_L |
|  | 44 | 0.000 |  | -2 | -52 | 30 | Cingulum_Post_L |
|  | 37 | 0.000 |  | -2 | 56 | 24 | Frontal_Sup_Medial_L |
|  | 19 | 0.000 |  | -42 | -18 | -4 | Temporal_Sup_L |
|  | 16 | 0.000 |  | 2 | 58 | -26 | Rectus_R |

Only clusters of 10 or more voxels are reported. Brain regions determined using the Automated Anatomical Atlas (aal) from WFU PickAtlas tool [2, 3].

Table S2. Common activation during Regulate>LookNegative present in LookNegative>LookNeutral.

| Contrast | Cluster size | $p_{FDR}$ | Peak t-value | x | y | z | Brain region |
| --- | --- | --- | --- | --- | --- | --- | --- |
| LookNegative >LookNeutral | 146 | 0.001 | 3.77 | -56 | -58 | 24 | Angular_L |
|  |  |  | 3.55 | -50 | -52 | 26 | Angular_L |
|  |  |  | 3.04 | -60 | -50 | 20 | Angular_L |
|  | 55 | 0.004 | 3.11 | 58 | 46 | 26 | Angular_R |
|  |  | 0.022 | 2.42 | 50 | 46 | 28 | Angular_R |

FDR>0.05. Brain regions determined using the Automated Anatomical Atlas (aal) from WFU PickAtlas tool [2, 3].

Table S3. Sample characteristics of age-matched groups

|  | HC  n=26  Mean SD | | OB  n=33  Mean SD t  *p* | | | |
| --- | --- | --- | --- | --- | --- | --- |
| Age (years) | 35.69 | 9.67 | 41.61 | 9.63 | -2.33 | **0.23** |

*HC* healthy control*; OB* obesity*; SD* standard deviation.

Table S4. DCM parameter estimates for the sample with age-matched groups

|  | Model Parameters | Effect size in Hz [90% CI] | Posterior Probability |
| --- | --- | --- | --- |
| Modulation by emotion regulation | **BMI** |  |  |
|  | dlPFC-amy | -0.007 [0, - 0.02] | 0.67 |
|  | dmPFC-amy | 0.006 [0, 0.02] | 0.67 |
|  | OFC-amy | 0.009 [0, 0.03] | 0.67 |

*BMI* body mass index; *dlPFC* dorsolateral prefrontal cortex; *amy* amygdala; *dmPFC* dorsomedial prefrontal cortex; *OFC* orbitofrontral prefrontal cortex.

**References**

1. Lang PJ, Bradley MM, Cuthbert BN. International affective picture system (IAPS): Instruction manual and affective ratings. 2008.

2. Maldjian JA, Laurienti PJ, Kraft RA, Burdette JH. An automated method for neuroanatomic and cytoarchitectonic atlas-based interrogation of fMRI data sets. Neuroimage. 2003;19:1233–1239.

3. Tzourio-Mazoyer N, Landeau B, Papathanassiou D, Crivello F, Etard O, Delcroix N, et al. Automated anatomical labeling of activations in SPM using a macroscopic anatomical parcellation of the MNI MRI single-subject brain. Neuroimage. 2002;15:273–289.
